# Objective structured clinical examination in radiology

**DOI:** 10.4103/0971-3026.63040

**Published:** 2010-05

**Authors:** Anurag Agarwal, Bipin Batra, AK Sood, Ravi Ramakantan, Satish K Bhargava, N Chidambaranathan, IK Indrajit

**Affiliations:** National Board of Examinations, NAMS Building Ansari Nagar, New Delhi 110 029, India; 1 Department of Radiology, KEM Hospital, Parel, Mumbai 400 012, India; 2 Department of Radiology and Imaging, University College of Medical Sciences (Delhi University) and associated GTB hospital, Delhi 110 095, India; 3 Department of Radiology and Imaging Sciences, Apollo Hospitals, Chennai 600 006, India; 4 Department of Radiodaignosis, Command Hospital (Air Force), Bangalore 560 007, India

**Keywords:** Assessment, radiology, OSCE

## Abstract

There is a growing need for introducing objective structured clinical examination (OSCE) as a part of radiology practical examinations in India. OSCE is an established, reliable, and effective multistation test for the assessment of practical professional skills in an objective and a transparent manner. In India, it has been successfully initiated and implemented in specialties like pediatrics, ophthalmology, and otolaryngology. Each OSCE station needs to have a pre-agreed “key-list” that contains a list of objective steps prepared for uniformly assessing the tasks given to students. Broadly, OSCE stations are classified as “manned” or “unmanned” stations. These stations may include procedure or pictorial or theory stations with clinical oriented contents. This article is one of a series of measures to initiate OSCE in radiology; it analyzes the attributes of OSCE stations and outlines the steps for implementing OSCE. Furthermore, important issues like the advantages of OSCE, its limitations, a strengths, weaknesses, opportunities, and threats (SWOT) analysis, and the timing of introduction of OSCE in radiology are also covered. The OSCE format in radiology and its stations needs to be validated, certified, and finalized before its use in examinations. This will need active participation and contribution from the academic radiology fraternity and inputs from faculty members of leading teaching institutions. Many workshops/meetings need to be conducted. Indeed, these collaborative measures will effectively sensitize universities, examiners, organizers, faculty, and students across India to OSCE and help successfully usher in this new format in radiology practical examinations.

## Introduction

There is a growing need for introducing objective structured clinical examination (OSCE) as a part of practical examinations for awarding professional degrees and diplomas in radiology in India. OSCE is a method of practical assessment. It is an established, reliable, and effective multistation test for the assessment of practical professional skills. Introduced by Hardin in 1979,[1] it has found increasing acceptance in various medical disciplines, chiefly due to its emphasis on objective assessment of students rather than subjective assessment. It has been successfully implemented by examination bodies in specialties like pediatrics, ophthalmology, and otolaryngology in India. OSCE is versatile in that “it can be and has been used for many levels of education, including undergraduate, postgraduate, continuing education, and licensure and certifying exams.”[2]

This review article seeks to demystify OSCE. It analyzes the attributes that make OSCE stations effective and outlines the list of measures for successful implementation of OSCE. The advantages of OSCE, its limitations (with suggestions for addressing the problems), and the timing of introduction of OSCE in radiology are covered in this article; a SWOT (strengths, weaknesses, opportunities, and threats) analysis of OSCE is also presented.

## Why is There a Need for OSCE?

The existing model of radiology practical examinations in India comprises of spots, long case, short cases, and table viva. Although this “time-honoured” assessment technique has been employed for long, there are a few problems that are intrinsic to this method. These have been analyzed in detail in many fora as well as in this journal earlier.[3–5] To recapitulate, the list of deficiencies includes an overall lack of objectivity, wide variability in assessing the skills of different students, and the inability to test the communication skills of students. The traditional format offers little useful feedback, allows favoritism to creep in, and does not permit standardization of the expertise of the different examiners in their role as “evaluators.” The traditional system also has limitations in predicting the future performance of radiology students.[6] Often, the ability to examine a patient, diagnose a disorder, and report it professionally is not analyzed objectively by examiners. To overcome these shortcomings, there is a need for introducing OSCE.

## Core Features of OSCE

The OSCE, comprising 10–20 stations, is a method of practical assessment. Depending on the specialty, the number of manned stations may vary from 4 to 10. In general, “each station presents part of a case or problem using simulated/standardized patients, slides, audio tapes, photographs, or laboratory reports, and requires examinees to perform a specific procedure, solve a problem, or record requested findings.”[2,7] In radiology, each station would focus on specific topics sourced from the prescribed curriculum.

Completion of a task within a single station involves one of the following: demonstrating a task to an examiner, providing verbal answers, or writing specific objective answers in a response sheet. Typically, a student spends 5 min within a station. The stations are concurrently run in a specific direction, which in practice means that approximately 10 students can be assessed in a period of 2 h.

## Types of OSCE Stations

OSCE stations are classified as “manned” or “unmanned” stations depending on the presence or absence of an examiner at the station. Of the total stations, no more than four OSCE stations can be manned.

*Manned* stations are often *procedure* stations. In a *procedure* station, the student performs a basic radiographic task, such as loading a film (radiography OSCE station) or demonstrates an examination technique, such as carrying out a USG examination of the abdomen in a patient (USG OSCE station). The performance of the examinee at any given station is judged against a standardized and pre-agreed “key-list” of steps, which serves as a guide to the examiner at the time of student assessment. These written steps are prepared prior to the exams and commence with patient care related inquiries and ethical questions, e.g., *does the student speak to the patient on entering the station? Does the student explain to the patient the procedure of USG? Does the student seek the permission of the patient before commencing the examination?* Or *Does the student provide a screen to maintain privacy during the examination?*[5,8]

In an *unmanned* station the student analyzes films and images, which indeed are the foundation of radiology as a specialty, or answers “context-specific” theory questions. Thus, in a *pictorial station,* the student analyzes a given film (radiographs, USG, CT scan, DSA, or MRI) and objectively answers specific questions. Likewise, in a *theory station* the student answers specific questions, with either paragraph answers or objective answers (“a,” “b,” “c,” “d,” “all of the above,” or “none of the above”).

Each station by itself is a “context-sensitive” objective assessment of an important topic from the radiology curriculum. Expectedly, this leads to the creation of different stations, with the overall emphasis being on conventional radiology, as is the case in exams conducted in USA and the UK. Foremost among the OSCE stations in radiology are the *basic OSCE stations* which would include conventional radiography and physics, CT scan, MRI, USG, and interventional radiology. *Advanced OSCE stations* may be subspecialty-based, e.g., chest radiology, musculoskeletal radiology, uroradiology, neuroradiology, etc. There may also be a combination of all modalities relevant to a given case. OSCE stations can be further evolved to create *specialized* *OSCE* *stations* dealing with emergency radiology,[9] prenatal sex determination test (PNDT), communication skills, reporting skills, ethical issues, and writing skills for article/dissertation publishing, all of which need consensus, validation, certification, and finalization.

## Measures Facilitating OSCE Implementation

OSCE stations should be designed to comprehensively test the professional skill of a student.[10] A brief outline of the parameters that support the successful implementation of OSCE is given in Table 1.

**Table 1:** Key issues in designing and implementation of OSCE in radiology[2,8,11,17]

Issues in designing station/s	Parameter	Requirements
Professional content	Assessment	It should assess students' understanding of theoretical concepts, observation, interpretation, and reporting skills
	Material	It should be from radiology curriculum and appropriate for outcomes being measured
	Pattern	The questions should be objective in nature
	Language	The language should be simple, clear, and easy to understand
	Difficulty level	Contents should not be too easy or too difficult
	Tasks steps	Sufficient number of tasks is necessary
	Time	For completion of a task at each station, adequate time should be factored in for each station
	Briefing	Students and examiners should be given clear briefing before conduct of OSCE
	Keys/answers	Pre-agreed “key-list” of steps mandatory for each station to ensure uniform assessment of students. The outlined steps should identify marks their relative weightage
	Scoring	Test scores is objectively based on adequate number of items
	Bias and errors	Scores should not be influenced by personal bias; strict adherence to pre-agreed “key-lists” for minimizing interexaminer error
Physical issues	Rooms	Rooms should have optimal lighting, space, air-conditioning, ventilation, and ambience
	Space	Adequate space should be provided to create the required number of cubicles or stations
	Number of stations	The number of stations may vary from 10 to 20, depending on marks allotted as a part of the total practical exams
	Layout	The number of stations should be clearly mentioned at entrance of examination center
	Timing	Time alloted to each station should also be clearly mentioned
	View boxes	Adequate number and optimal lighting of view boxes will be required
	Stationery	The examiners should be provided with questions, key sheet, answers, allotted marks, detailed instructions, etc.
	Marking	No negative marks are awarded in this format of examination
	Movement	Students should proceed sequentially in only one direction along numbered stations
	Calling bell/buzzer	Calling bell/buzzer should be heard across all stations. It should be heard at the start and end of a station

Broadly, there are two issues that need to be addressed. First and foremost is the design of the professional content of radiology OSCE stations.[11] An important intention in designing OSCE stations for radiology is that the assessment should be clinically oriented. The student should identify the abnormality/abnormalities, methodically correlate the data, and extract the clinical content of a given radiological study to make a meaningful contribution toward the correct management of a given case. The selection of objective tasks at each OSCE station should assess a student's understanding of concepts, observation, interpretation, and reporting skills in radiology. The language used at the station should be simple, clear, and easy to understand. During designing of a station, adequate time should be factored in for completion of a task at the station.

Importantly, a pre-agreed “key-list” is created by examiners for each station. It consists of an outline or a list of objective steps, which will ensure uniformity in the assessment of students. At each station, the marks awarded are objective and have a relative weightage factor. How close to or how far away a student's answer is as compared to the examiner-prepared, pre-agreed “key-list” determines the performance at any given station.

The other important issue is the physical design of OSCE stations. Here attention must be given to details such as the layout and signposting, lighting and air-conditioning of rooms, the number of OSCE stations, the status of the view-boxes, stationery, etc.

## Advantages of the OSCE Format

The OSCE format has numerous advantages. “OSCE is applicable to any area of medical education.”[12] The entire examination is objective and promotes transparency.[13] A large number of students can be evaluated within a short time. It encourages increased interaction between the examiner and students.[14] It facilitates a convenient integration of teaching and evaluation. The variability posed by assessment of students on dissimilar patients, different cases, and assorted topics is drastically trimmed down. Similarly, the variability that exists among different examiners is bypassed when many students are assessed using a standardized format, which advantageously leads to objectivity in assessment.[8] OSCE has been successful in removing biases associated with traditional examination systems.

Once introduced in the syllabus, practice OSCEs are an inevitability. Students can use these practice OSCEs to identify stations where their performance is suboptimal and attempt to improve their skills in that area. OSCE thus “identifies areas of weakness in the curriculum and/or teaching methods” and thereby improves educational effectiveness.[6] Morag *et al.* have shown that “OSCE may be useful to uncover deficits in individuals and groups beyond the ones detected with traditional clerkship evaluations and provides guidance for remediation.”[6] Likewise, a study by Hamann *et al.* showed that in practice OSCE sessions, the “station scores identified specific content that needs improved teaching.”[15] Literature further adds that “OSCE examinations are more likely to measure other qualities such as problem-solving abilities, critical thinking, and communication skills.”[16]

With increased acceptance in the academic fora in India and the availability of interesting radiology cases in teaching institutions, there is ample scope for creating OSCE question-and-answer banks featuring a large storehouse of educational material. OSCE is able to provide truthful, honest, and genuine feedback to the candidates, clearly identifying their strengths and weaknesses.

## Difficulties with the OSCE Format and Overcoming Them

OSCE's strength lies in its unique format, which enhances objectivity and transparency. However, a few problems are inevitably present. To begin with, many first timers find OSCE to be a “strong anxiety-producing” experience.[17] However, it must be kept in mind that this is also the case in the other existing formats that include theory and/or practical exams.

A reported difficulty experienced by students is the “inadequacy of time” for expected tasks at some stations.[17] Where this is a genuine problem, examiners should design stations such that there is adequate time per station. Additionally, at the time of setting up the OSCE, the group of examiners can decide to do away with “time-wasting” or subjective questions. Reusing OSCE stations has its share of implications but the problems can be eventually overcome.[18] A SWOT analysis of OSCE is given in Table 2.

**Table 2:** SWOT analysis of OSCE

**Strength**	**Weakness**
Objective	Costly
Authentic	Time-consuming in preparation
Reliable and valid	Requires large area/space
Provide candidates feedback	
Ability to evaluate in real time	
Uniformity in assessment	
Overcomes bias of traditional examination	
Ensures wide coverage of curriculum	

**Opportunity**	**Threat**

To replace traditional examination system	Opposition to the idea of revamping traditional system with OSCE
Implementation successful and widely accepted in other specialties like pediatrics and ophthalmology	Delay in active participation and brainstorming, which is mandatory for OSCE
Usher in objectivity in assessment at the expense of subjective	Negative mindset of faculty members due to a feeling of threat

For the organizers at exam centers, the examination may be costly[19] and effort-consuming. This is also true when designing and preparing any format of practical exams (spotters, MCQs, etc.), and it is encouraging to find that other specialties in India have overcome these initial difficulties; literature reports that “the new OSCE format posed no organisational problems”[20] and that “subsequent exams will not require the same degree of administrative load.”[21]

OSCE in radiology requires more inputs from the faculty members of leading teaching institutions. A series of workshops/meetings need to be organized so that the OSCE can be validated, certified, and finalized before it is used for examination purposes. This entails multiple and repeated brainstorming sessions with the active participation and contribution of the faculty members. This may be time consuming and, most importantly, will call for determination and zeal on the part of the faculty members to switch over from the traditional method of examinations to the more rational, objective, and methodical OSCE.

To overcome these difficulties, there is a compelling need for all educators in radiology to plan a series of inclusive, constructive steps. An expert body empanelled under the aegis of the Indian College of Radiology and Imaging (ICRI), National Board of Examinations (NBE), leading universities, and reputed teaching institutions under the Medical Council of India (MCI) across the country should commence designing OSCE tests in radiology. The question banks created for different OSCE stations should be implemented gradually over time. University and governing bodies should direct various hospitals and teaching institutes and centers to compulsorily introduce and conduct OSCE, over a period of a year. This will enable examiners, organizers, faculty, and students across India to become familiar with the OSCE format. This will be the key to the initiation and successful implementation of OSCEs in radiology.

## A few Special Issues in Designing OSCE Stations in Radiology

Communication and reporting skills are important aspects of radiology[22–23] that are not assessed in traditional radiology practical examinations, but are extremely important in clinical practice. It is well known that inadequate and faulty communication of findings may lead to preventable medical errors, with morbidity and mortality of upto 20%.[24–25] OSCE provides an opportunity to assess these neglected skills by allowing the creation of specifically designed stations for this purpose. Similarly OSCE “provides a means of assessing radiology resident reporting skills,” which is yet another neglected area that is not comprehensively assessed in traditional exams.[24]

Radiology as a specialty deals with the acquisition and analysis of images. In recent times, digital images have become the mainstay of all modalities, including conventional radiography, leading to a new form of workflow. An “electronic OSCE” may create the opportunity to assess students using digital images with the help of computers and projectors[26] and possibly, even the Web[27] or handhelds. Although still in its infancy, an initial experience with “electronic OSCE” has demonstrated “user-friendliness, interactivity, and navigation facilities.”[28] Further, “this form of assessment is considered to be cost-effective in terms of staff and equipment resources' and, besides, appears to be preferred by students.”[28]

## Feasibility and Timing of Introduction of OSCE in Radiology

OSCE is now a part of practical examinations in many medical disciplines. In the prevailing radiology model, practical assessment in MD, DMRD, and DNB examinations comprises varyingly of assessment formats such as spots, long cases, short cases, table vivas, and MCQs. The introduction of OSCE as a new format will either require doing away with one or more of the existing formats[29] or OSCE could be an additional format. It must be remembered that all exams face limitations of time and space. In view of this, replacing rather than adding OSCE may be more feasible when incorporating it in the assessment formats. In view of the fact that spots and long cases are “time-tested” methodologies, perhaps it would be practical to replace one or both of the table vivas with OSCE.

The final model may be arrived at once different exam-conducting bodies gain experience and learn during the process of assessment evolution. Despite it being a tall order, OSCE needs to be implemented. Finally, it must be reiterated that the OSCE technique does not replace the existing model of practical examinations, but augments it effectively by being transparent and enhancing objectivity.

## Summary and Conclusion

OSCE is an examination format that uses a contextual format at multiple stations. It facilitates assessment of core competence and contemporary professional skills in several medical disciplines in an objective and a transparent manner. In India, it has been successfully initiated and implemented by examination bodies in specialties like pediatrics, ophthalmology, and otolaryngology.

As regards radiology, the time may have come for incorporating OSCE as a part of the practical examination. This will require several collaborative measures such as (a) active participation and contribution from the academic radiology fraternity, (b) inputs from faculty members of leading teaching institutions, (c) organization of a series of workshops/meetings, (d) sequencing the critical process of validation, certification, and finalization before the use of OSCE in radiology examinations.
